# An Infrequently Encountered Cause of Hemoptysis

**DOI:** 10.7759/cureus.45954

**Published:** 2023-09-25

**Authors:** Prakash Banjade, Yasoda Rijal, Asmita Itani, Becky X Lou, Munish Sharma

**Affiliations:** 1 Internal Medicine, Manipal College of Medical Sciences, Pokhara, NPL; 2 Internal Medicine, Institute of Medicine Tribhuvan University, Kathmandu, NPL; 3 Internal Medicine, GP Koirala National Center for Respiratory Diseases, Tanahun, NPL; 4 Pulmonary, Critical Care and Sleep Medicine, Baylor Scott & White Medical Center - Temple, Temple, USA; 5 Pulmonary and Critical Care, Baylor Scott & White Medical Center - Temple, Temple, USA

**Keywords:** pulmonary apoplexy, valvulopathies, hemoptysis, mitral stenosis, cardiogenic shock

## Abstract

Mitral stenosis (MS) is not a common entity in modern-day medicine, especially in developed countries, as the most common etiology is still rheumatic fever. MS can present mainly with a wide range of cardiac symptoms. However, infrequently, MS can cause extra-cardiac symptoms as well. We present a case report of a patient with severe bioprosthetic mitral valve stenosis with intermittent hemoptysis and cardiogenic shock. We aim to report this case to remind clinicians about this uncommon but significant cause of hemoptysis. This case report also emphasizes the importance of utilizing a team approach while treating patients with severe MS, especially if they have serious complications that could be life-threatening. We also aim to add to the current literature by reporting this case.

## Introduction

Mitral stenosis (MS) is a type of heart valve disease that occurs when the mitral valve’s opening narrows. The primary cause of this condition is rheumatic fever [1]. The incidence of rheumatic fever is decreasing in developed countries. The prevalence of rheumatic MS is higher in developing countries compared to the United States [1]. Patients with MS exhibit symptoms caused by reduced blood flow over the valve, leading to congestion and higher pressures in the pulmonary circulation. Hemoptysis is a less frequently encountered symptom and occurs when pulmonary vessels rupture due to increased pressure in those vessels. Hemoptysis in MS can manifest in different ways, such as sudden bleeding (known as pulmonary apoplexy) or coughing up pink frothy sputum due to pulmonary edema [2]. Severe MS can present with cardiogenic shock as well [3]. We present a case of severe bioprosthetic mitral valve stenosis who presented to our hospital with cardiogenic shock and intermittent hemoptysis.

## Case presentation

**Figure 1:**
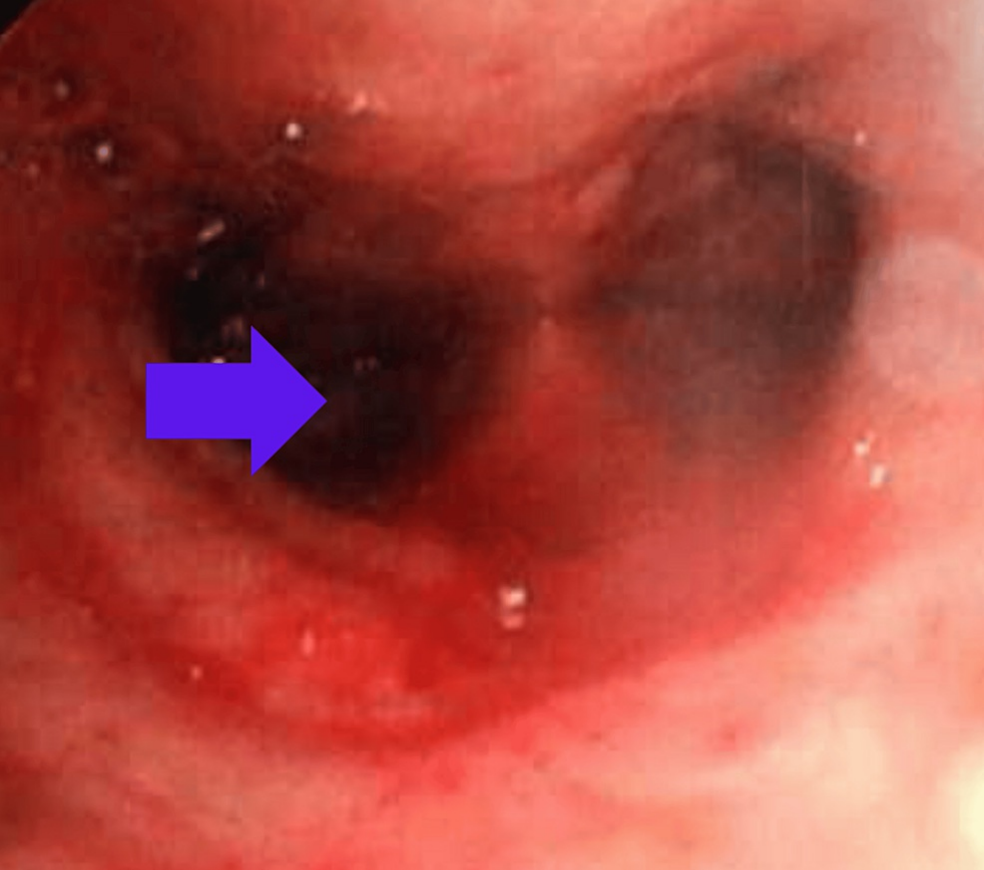
Bronchoscopy image showing bleeding in bilateral main stem bronchi (right more than left). The blue arrow shows the right mainstem bronchi entry.

An 84-year-old Latin American female patient, with a medical history of essential hypertension, coronary artery disease status post coronary artery bypass graft (CABG), and bioprosthetic mitral valve replacement for unknown duration, was referred to our hospital primarily due to worsening shock. She was initially admitted to an outside facility with a complaint of generalized weakness and shortness of breath for five days. She was resuscitated with 3 L of crystalloid and was started on norepinephrine, vancomycin, piperacillin/tazobactam, and hydrocortisone for presumed septic shock. As her condition continued to deteriorate, she was referred to us. On arrival at our facility, her initial assessment revealed a noninvasive blood pressure of 94/54 mmHg, heart rate of 108 beats per minute, normal sinus rhythm, respiratory rate of 22 breaths/min, oxygen saturation of 93% on 6 L per minute of oxygen, and temperature of 98.8 F. She was alert and awake; respiratory examination revealed bibasilar crackles, and cardiac examination showed S1S2 with a mid-diastolic murmur. On further review of her history, the patient had been having intermittent episodes of hemoptysis for two months before admission. Hemoptysis started with a small amount of blood-streaked sputum, which gradually progressed. Her primary pulmonologist had evaluated her as an outpatient, and she had undergone a bronchoscopy (Figure 1) with bronchoalveolar lavage (BAL) and transbronchial biopsy of the right lung. Cytology specimens from both BAL and transbronchial biopsy did not reveal malignant cells. The acid-fast bacilli stain was negative. Bacterial and fungal cultures were also negative. A transbronchial biopsy pathology sample showed minute fragments of bronchial mucosa with focal anthracosis. No neoplasm was identified. Her autoimmune workup was also negative. She was treated with steroids and antibiotics empirically for a week, but her intermittent hemoptysis did not resolve. After the results of BAL and transbronchial biopsy, steroids, and antibiotics were discontinued.

**Figure 2:**
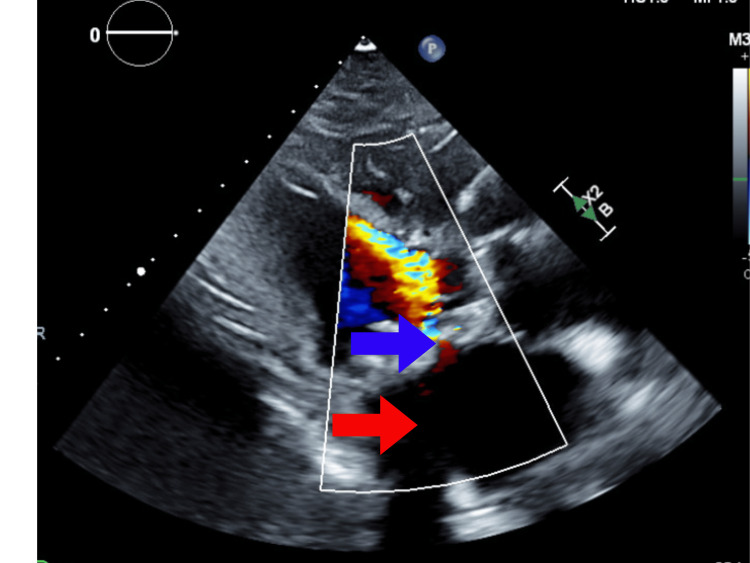
Preoperative echocardiogram showing mitral valve stenosis (blue arrow) and dilated left atrium (red arrow).

**Figure 3:**
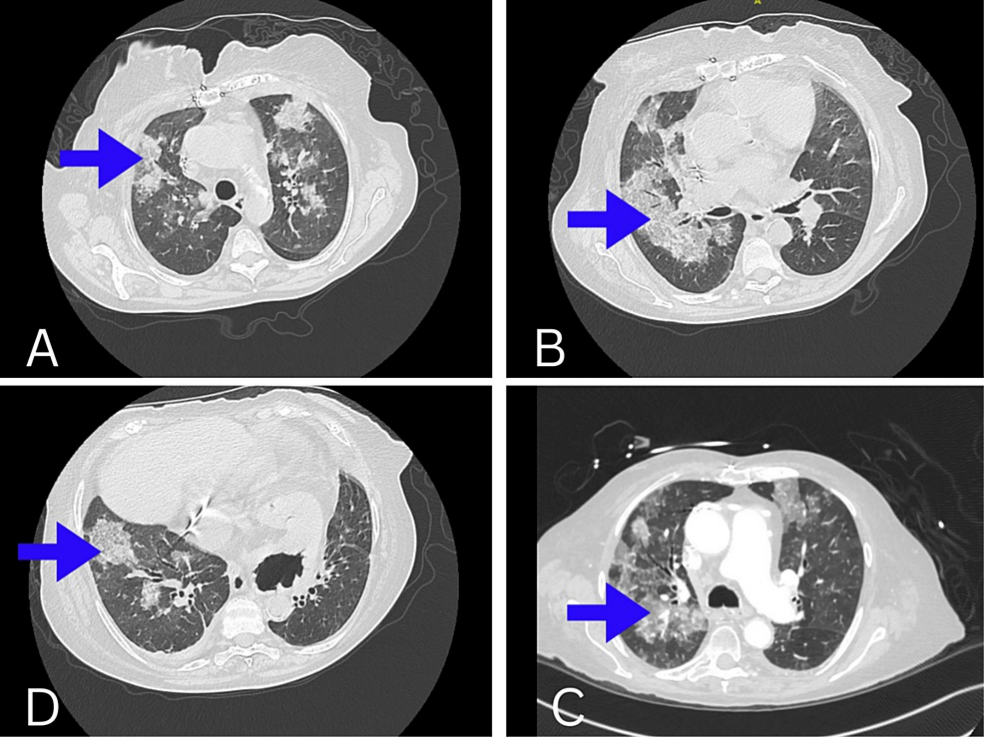
Preoperative computed tomography scan chest images showing ground-glass opacity in the upper lobe (A), middle lobe (B, C), and lower lobe (D).

After admission to our facility, echocardiography (Figure 2) showed a left ventricle ejection fraction (LVEF) of 50% with a left ventricular outflow tract systolic volume index of 39 ml/m^2^. Tricuspid annular plane systolic excursion (TAPSE) was 1.3 cm. The right ventricular peak systolic velocity (RVS’) was 5.2 cm/s. The left atrium was severely dilated. There was a bioprosthetic mitral valve with severe prosthetic mitral valve stenosis. The mean gradient was 20 mmHg. The mitral valve area by velocity time integral (VTI) was 0.4 cm^2^. Subsequently, she was diagnosed with cardiogenic shock secondary to severe MS. Right and left heart catheterization were performed. Right atrial pressure was 25/26 (21) mmHg, pulmonary artery pressure was 78/38 (56) mmHg, capillary wedge pressure was 55 mmHg, Fick cardiac output was 3.10 L/min, and Fick cardiac index was 2.03 L/min/m^2^. Analysis of the mitral valve showed a mean gradient of 28.85 mmHg, flow of 40.55 ml/s, area of 0.20 cm^2^, and area index of 0.13 cm^2^/m^2^. Computed tomography (CT) chest showed scattered bilateral ground glass and consolidative opacities bilaterally (Figure 3).

**Figure 4:**
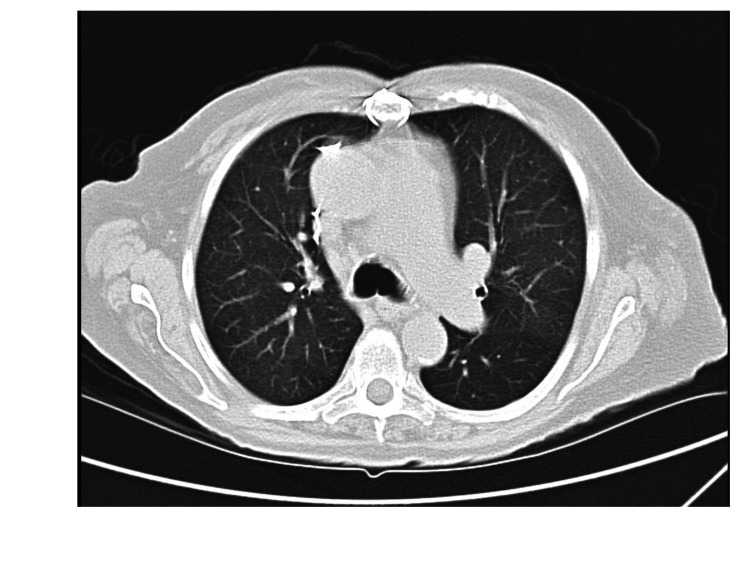
Computed tomography chest three months after mitral valve surgery showing resolution of prior scattered bilateral ground glass and consolidative opacities.

**Figure 5:**
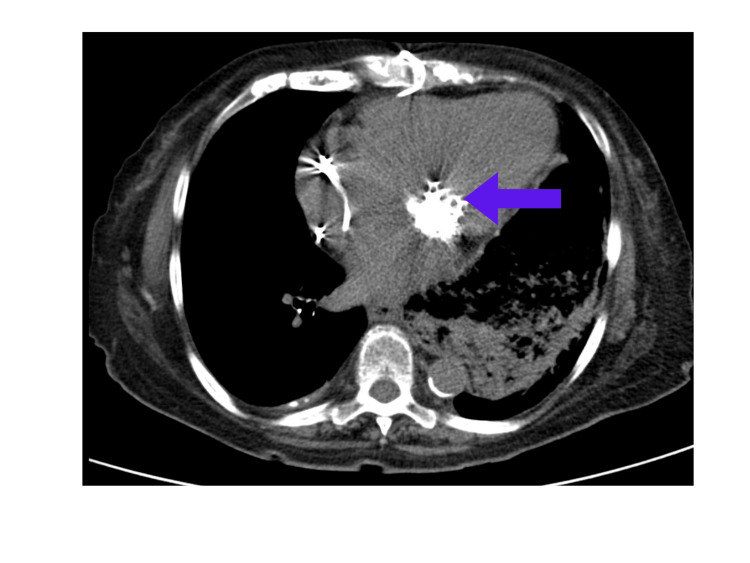
Computed tomography chest three months after mitral valve surgery. The blue arrow shows the replaced mitral valve.

**Figure 6:**
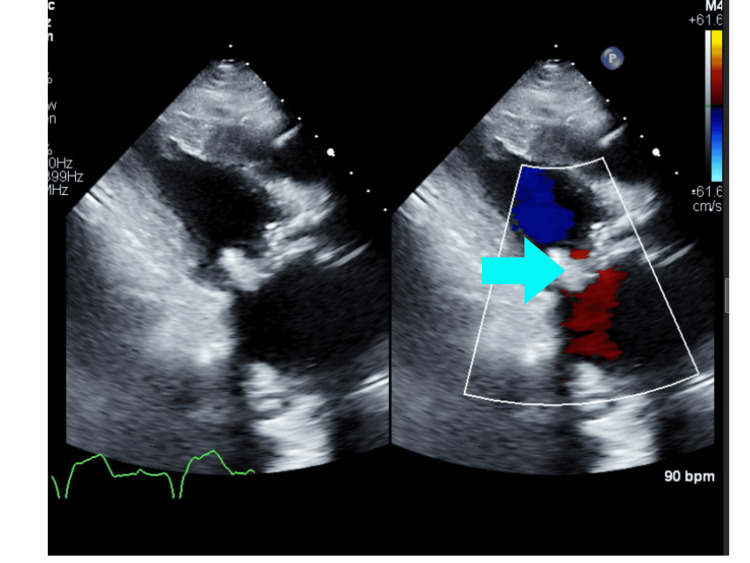
Echocardiography three months after surgery showing resolution of severe MS.

Cardiothoracic surgery and interventional cardiology were consulted for the management of severe MS. The patient underwent transcatheter mitral valve replacement (TMVR) at our hospital via a transfemoral route with implantation of a SAPIEN 3 Ultra Resilia, a 26 mm valve. An aggressive diuresis was also done. Her shock resolved postoperatively. Her oxygen requirement decreased to 2 L per minute within a few days post-valve replacement. Her hemoptysis resolved completely. CT chest and echocardiography were performed three months after discharge. CT scan showed complete resolution of prior scattered bilateral ground glass and consolidation opacities bilaterally with an intact replaced mitral valve (Figures 4, 5). Echocardiography showed a 26 mm SAPIEN 3 Ultra Resilia prosthetic mitral valve-in-valve, with no evidence of prosthetic mitral valve obstruction and significant improvement of flow patterns (Figure 6). The mean gradient was 6 mmHg at a heart rate of 80 beats per minute. Doppler velocity index was 1.8.

## Discussion

Our patient presented with cardiogenic shock and intermittent hemoptysis secondary to severe bioprosthetic mitral valve stenosis. Hemoptysis, the coughing up of blood from the lower respiratory tract, is reported in approximately 0.1% of non-hospitalized and 0.2% of hospitalized patients annually [4]. Hemoptysis can occur in various underlying pathologies, and the reasons behind it are still unclear in almost half of all occurrences. Commonly recognized causes include infectious and inflammatory respiratory illnesses (25.8%) and malignancy (17.4%) [4]. Diagnosing and treating massive hemoptysis can be challenging, even though over 90% of cases tend to resolve on their own [5]. Although uncommon, cardiovascular issues, such as pulmonary edema/MS (4.2%) and pulmonary artery embolism (2.6%), are clinically significant causes of hemoptysis [6]. The primary cause of MS globally is rheumatic fever [1]. Calcification of the mitral valve leaflets and congenital heart disease can also cause MS, but they are less frequent causes. Rheumatic disease occurrence is declining in developed nations, with an estimated rate of 1 in 100,000. Nonetheless, the prevalence of this disease is greater in developing countries than in the United States [1]. The bioprosthetic valve is more prone to structural valve degeneration, resulting in obstruction and stenosis, as compared to the native valve [7]. The typical size of the mitral valve opening is 4-5 cm^2^. However, in cases of MS, the orifice becomes narrower and restricts the free movement of blood from the left atrium to the left ventricle. This creates a pressure gradient between the two chambers, which is added to the left ventricular diastolic pressure. As a result, the left atrial pressure increases, leading to enlargement of the left atrium and, ultimately, pulmonary congestion. Severe MS, characterized by a mitral valve area of less than 1 cm^2^, significantly limits the flow and decreases the left ventricular output [8]. Mortality from severe MS and cardiogenic shock can reach close to 25% if it is not treated accordingly [3]. Timely diagnoses and appropriate intervention, such as valve replacement or percutaneous balloon mitral valvuloplasty (PBMV), are imperative for favorable patient outcomes. The most commonly encountered occurrence is an underlying MS affected by septic and hypovolemic shock, which may trigger cardiogenic shock [3].

MS has been reported to cause five different forms of hemoptysis: pulmonary apoplexy, congestive hemoptysis, pulmonary edema, winter bronchitis, and pulmonary infarction [9]. Hemoptysis is attributed to sudden dilatation of pulmonary capillaries and diapedesis of red blood cells into pulmonary alveoli, rupture of necrotic vessels involved in the acute rheumatic process, shunting of blood from pulmonary to bronchial veins, or bleeding from the pleurohilar veins [10]. However, hemoptysis due to MS is rare in contemporary practice because of early recognition and intervention, and its presence usually suggests an advanced disease [9]. The reported case was also diagnosed with severe MS, which resulted in intermittent hemoptysis. In our patient, hemoptysis might have been caused by increased pressure in the left atrium, subsequently causing dilatation of pulmonary capillaries.

The physical examination can provide valuable diagnostic information. When a disease is in advanced stages, the pulse pressure may decrease, indicating a reduction in stroke volume. The distance between S2 and the opening snap provides a reliable indication of the severity of MS. An interval of less than 0.08 seconds between S2 and the opening snap usually suggests severe disease. A low-pitched rumble of the mitral valve follows the opening snap, which may be punctuated with presystolic accentuation when the patient is in sinus rhythm [8]. The clinicians must conduct a comprehensive examination of the patient. While the electrocardiogram (ECG) and chest radiograph can help give diagnostic clues in MS, the echocardiogram is currently considered the primary diagnostic tool [8]. Sometimes, when the information obtained from noninvasive procedures and the patient’s clinical condition does not match, cardiac catheterization determines the severity of MS. This procedure involves measuring cardiac output and transvalvular gradient to calculate the valve area. This helps to reassess the extent of stenosis [11]. Medical treatments to stabilize patients with MS include optimal ventilatory and inotropic support. When a patient’s condition remains unstable despite treatment of precipitating factors, emergent mechanical relief of MS should be done as soon as possible with either PBMV or surgery [3].

## Conclusions

This case report highlights a clinical scenario where severe MS caused a combination of hemoptysis and cardiogenic shock. Hemoptysis, as one of the main presenting symptoms due to severe MS of the bioprosthetic valve, is not frequently encountered in modern-day medicine. Especially in developed countries, it may not be a common presentation. Recognizing these symptoms promptly and quickly intervening with a multidisciplinary approach is crucial for favorable patient outcomes.
